# Identification of key amino acid residues in the hTGR5–nomilin interaction and construction of its binding model

**DOI:** 10.1371/journal.pone.0179226

**Published:** 2017-06-08

**Authors:** Takashi Sasaki, Moeko Mita, Naho Ikari, Ayane Kuboyama, Shuzo Hashimoto, Tatsuya Kaneko, Masaji Ishiguro, Makoto Shimizu, Jun Inoue, Ryuichiro Sato

**Affiliations:** 1 Food Biochemistry Laboratory, Department of Applied Biological Chemistry, Graduate School of Agricultural and Life Sciences, University of Tokyo, Tokyo, Japan; 2 Department of Applied Life Sciences, Niigata University of Pharmacy and Applied Life Sciences, Higashijima, Akiha-ku, Niigata, Japan; 3 Nutri-Life Science Laboratory, Department of Applied Biological Chemistry, Graduate School of Agricultural and Life Sciences, University of Tokyo, Tokyo, Japan; 4 AMED-CREST, Japan Agency for Medical Research and Development, Chiyoda-ku, Tokyo, Japan; Russian Academy of Medical Sciences, RUSSIAN FEDERATION

## Abstract

TGR5, a member of the G protein-coupled receptor (GPCR) family, is activated by bile acids. Because TGR5 promotes energy expenditure and improves glucose homeostasis, it is recognized as a key target in treating metabolic diseases. We previously showed that nomilin, a citrus limonoid, activates TGR5 and confers anti-obesity and anti-hyperglycemic effects in mice. Information on the TGR5–nomilin interaction regarding molecular structure, however, has not been reported. In the present study, we found that human TGR5 (hTGR5) shows higher nomilin responsiveness than does mouse TGR5 (mTGR5). Using mouse–human chimeric TGR5, we also found that three amino acid residues (Q77^ECL1^, R80^ECL1^, and Y89^3.29^) are important in the hTGR5–nomilin interaction. Based on these results, an hTGR5–nomilin binding model was constructed using in silico docking simulation, demonstrating that four hydrophilic hydrogen-bonding interactions occur between nomilin and hTGR5. The binding mode of hTGR5–nomilin is vastly different from those of other TGR5 agonists previously reported, suggesting that TGR5 forms various binding patterns depending on the type of agonist. Our study promotes a better understanding of the structure of TGR5, and it may be useful in developing and screening new TGR5 agonists.

## Introduction

TGR5, also known as G protein-coupled bile acid receptor 1 (GPBAR1), is a class A G protein-coupled receptor (GPCR) comprising seven transmembrane helices (TM), three extracellular loops (ECL), and three intracellular loops (ICL). TGR5 recognizes bile acids (BA) as its endogenous ligands [1, 2], and ligand-bound TGR5 activates the Gαs-subunit of heterotrimeric G proteins, resulting in the activation of adenylate cyclase and an increase in intracellular cAMP concentration. Subsequently, cAMP promotes a protein kinase A (PKA)–cAMP response element-binding protein pathway, which regulates diverse metabolic processes independently of the farnesoid X receptor, a nuclear bile acid receptor [1–3].

TGR5 is expressed in different tissues and organs, including brown adipose tissue (BAT), small intestine and skeletal muscle [4]. Watanabe et al. showed that TGR5 stimulates energy expenditure through the induction of a cAMP-dependent thyroid hormone-activating enzyme, type II iodothyronine deiodinase (Dio2), in BAT and prevents high-fat-diet-induced obesity and insulin resistance [5]. It has also been recently shown that BA promotes mitochondrial uncoupling and Dio2 expression in human brown adipocytes, along with increased BAT energy expenditure in women [6]. Furthermore, TGR5 activation induces the release of glucagon-like peptide-1 (GLP-1) by enteroendocrine L cells and the colon, thereby protecting against insulin resistance and improving glucose tolerance in obese mice [7–10]. Moreover, the anti-obesity and anti-diabetic effects of vertical sleeve gastrectomy are mediated in part by TGR5 activation, altering bile-acid concentration and composition in mice [11, 12]. Because of such beneficial effects on metabolism, TGR5 is recognized as an important target in treating metabolic diseases [13].

Recently, a number of novel TGR5 agonists were described [7, 14–18]. For example, 6alpha-ethyl-23(S)-methyl-cholic acid (EMCA, INT-777), a semisynthetic cholic acid derivative, works as a selective TGR5 agonist and regulates glucose homeostasis by promoting GLP-1 release from enteroendocrine L cells [7]. In addition, oleanolic acid, betulinic acid, and ursolic acid are TGR5 agonists found in plants [14, 15]. We previously showed that nomilin, a limonoid extracted from Citrus spp., exhibits TGR5 agonist activity and that dietary nomilin suppresses high-fat-diet-induced obesity and hyperglycemia in mice [16]. However, information regarding the structure of molecules involved in the binding between TGR5 and its agonists remains unclear [17, 19, 20], despite its importance in developing effective agonists.

In the present study, we planned to determine key amino acid residues in the TGR5–nomilin interaction and identify the molecular features of nomilin necessary for it to be recognized as a TGR5 agonist. We found that human TGR5 (hTGR5) shows higher nomilin responsiveness than does mouse TGR5 (mTGR5) regardless of their high homology. Considering this, several mouse–human TGR5 chimeras were developed to ascertain the critical region for nomilin response. Point mutation studies on the identified region revealed three key hTGR5 residues essential for hTGR5–nomilin interaction: Q77^ECL1^, R80^ECL1^, and Y89^3.29^. On the basis of these results, we constructed the hTGR5–nomilin binding model, which suggests that the molecular structure of nomilin is important to its being an hTGR5 agonist. Obacunone, a limonoid with a molecular structure similar to nomilin, also exhibited semi-specific hTGR5 ligand activity depending on the three amino acid residues mentioned earlier. Notably, the binding pattern between hTGR5 and these limonoids was different from that between hTGR5 and taurolithocholic acid (TLCA) and INT-777 [19, 20]. These findings provide useful structural information for the future development of new TGR5 agonists.

## Materials and methods

### Compounds

Nomilin was purchased from Tokyo Chemical Industry Co., obacunone was purchased from ChromaDex, and TLCA and limonin was purchased from Sigma.

### Plasmid constructs

The pCRE-Luc reporter plasmid, which contains four copies of the consensus sites of CRE, was purchased from Agilent Technologies. Mouse–human TGR5 chimeras (1 and 2) were constructed by using the primer set shown in Table A in S1 File and were inserted into the p3×FLAG-CMV-7 vector. Other TGR5 chimeras (except for Chimera-6) and point-mutated TGR5 constructs were synthesized using the overlap-extension PCR method. Primer sets for Chimeras 3–9 and mutation constructs are described in Table B-F in S1 File.

### Cell culture

HEK293 cells obtained from ATCC were cultured at 37°C under 5% CO2 in Dulbecco’s Modified Eagle’s Medium (DMEM) and supplemented with 100 units/mL penicillin, 100 μg/mL streptomycin, and 10% (v/v) fetal bovine serum (FBS).

### Luciferase assay

HEK293 cells were plated in 12-well plates at a density of 1.0 × 10^5^ cells/well. Twenty hours later, the cells were transfected with pCRE-Luc, p3×FLAG-CMV-TGR5, and pEF-β-galactosidase (100 ng/well each) using the calcium phosphate method. After 4 h of incubation, the medium was replaced with DMEM, which contained 10% dextran charcoal-stripped FBS. Twenty-four hours after transfection, the cells were treated with the previously mentioned compounds and control vehicle (DMSO). After 5 h, the cells were then incubated with lysis buffer (25 mM Tris-phosphate [pH 7.8], 2 mM dithiothreitol [DTT], 2 mM trans-1,2-Diaminocyclohexane-N,N,N, N′-tetraacetic acid [CDTA], 10% glycerol, and 1% Triton X-100) on ice, and the luciferase and β-galactosidase activity were measured. The luciferase activity values were divided by those of β-galactosidase to obtain normalized luciferase values.

### Molecular modelling of hTGR5 and ligand–receptor complexes

A 3D molecular model of hTGR5 was constructed based on metarhodopsin structure [21], an activated rhodopsin photointermediate (PDB ID:3PQR) which has a close structural similarity to an activated form of adrenergic receptor (PDB ID:3P0G) [22], as previously reported on 3D structural model of GPCR [23]. The activated form of GPCRs has only subtle structural difference from the inactive form although the active form has an outward movement of the cytoplasmic end of transmembrane 6. Thus, the binding site for agonists (active form) may have a similar to that of antagonists (inactive form). A homology alignment between rhodopsin and other GPCRs, such as adrenergic and dopaminergic receptors, was developed using homology module installed in Insight II. Then, the alignment was modified to conserve the highly-conserved residues found in TM regions (TM1:Asn32, TM3:Arg110, TM4:Trp146, TM5:Pro176, TM6:Pro239, TM7:Pro277) except for TM2 where highly conserved Asp residue is not found in the TGR5 sequence. Consequently, Ala60 was aligned for the Asp residue in the rhodopsin sequence. The seven TM regions and the extracellular and intracellular loops of hTGR5 were constructed using the homology modeling method, in which deletion and insertion sites are located in only loop structures. The initial structure model was energy-minimized with molecular mechanics and then optimized with molecular dynamics calculations equilibrating for 1ns at 273K. The ligand-binding space within the TM regions was examined using the binding-site module installed in Insight II software (Accelrys Inc., San Diego, USA). Thus, the fairly rigid ligands were docked in the putative binding sites navigated with the results of the present mutational experiments in two modes, in which a furan ring was located at the extracellular end (present model) and an upside-down mode, as described in the previous docking study on bitter taste receptor model [24].

The initial complex structures were energy-minimized and then optimized using molecular mechanics and dynamics calculations, tethering the α-carbon of the main chain in the TM regions at the initial positions. The final complex structures were then structure-optimized in the lipid bilayer model installed in AMBER software.

### Statistical analysis

All data are presented as mean ± standard deviation (SD). Two-tailed unpaired Student’s t-tests and one-way ANOVA (Tukey’s post hoc test) were used to determine p-values. Statistical significance was set at p < 0.05.

## Results

### Nomilin exhibits different agonist activity between hTGR5 and mTGR5

**Fig 1:**
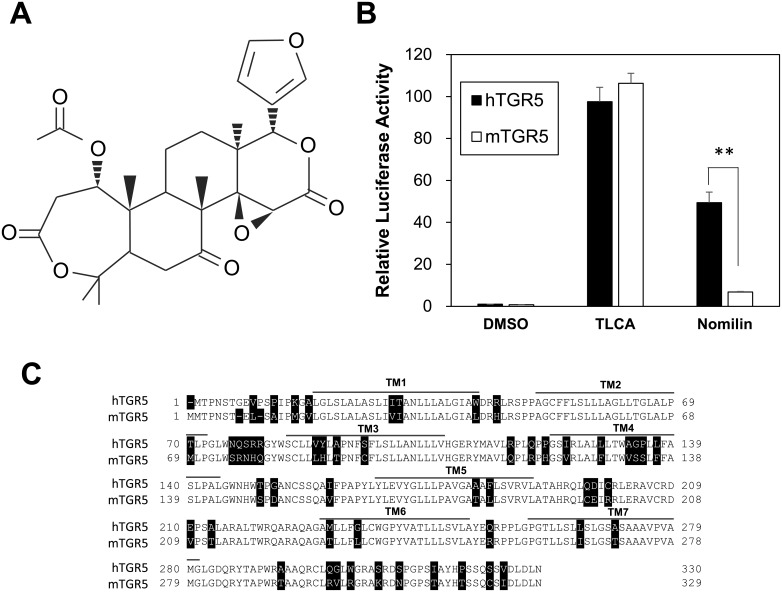
Differences between hTGR5 and mTGR5 with respect to nomilin response and amino acid sequences. (A) Structural representation of nomilin. (B) HEK293 cells were transfected with the CRE-driven luciferase reporter plasmid and the hTGR5/mTGR5 expression plasmid. After transfection for 24 h, the cells were treated with TLCA (positive control) and nomilin (100 μM each) for another 5 h. Then, a luciferase reporter assay was performed, normalizing against β-galactosidase activity. The promoter activity of hTGR5/DMSO was set at 1 (n = 3). (C) Amino acid-sequence alignment and TM domain of the hTGR5 and mTGR5; unconserved amino acids are shown in inverted color. Significant differences were analyzed using one-way ANOVA (Tukey’s post hoc test); **p < 0.01. The values represent the mean ± SD.

We previously reported that nomilin, which is a limonoid frequently found in citrus plants (Fig 1A), has the potential to activate hTGR5 and that dietary nomilin suppressed diet-induced obesity and hyperglycemia in mice [16]. Despite the favorable effect of nomilin in mice, we found that nomilin exhibits considerably weaker agonist activity for mTGR5 than it does for hTGR5, as shown by using the cAMP response element (CRE)-luciferase reporter assay system in HEK293 cells. In contrast, TLCA (a positive control) activated hTGR5 and mTGR5 equivalently (Fig 1B). Because mTGR5 and hTGR5 share a homology of >80% in the amino acid sequence, we hypothesized that 55 heterologous amino acid residues (black-and-white reversal amino acid residues in Fig 1C) contain key amino acid residues for the hTGR5–nomilin interaction.

### Ascertaining the critical region of hTGR5 for nomilin response

**Fig 2:**
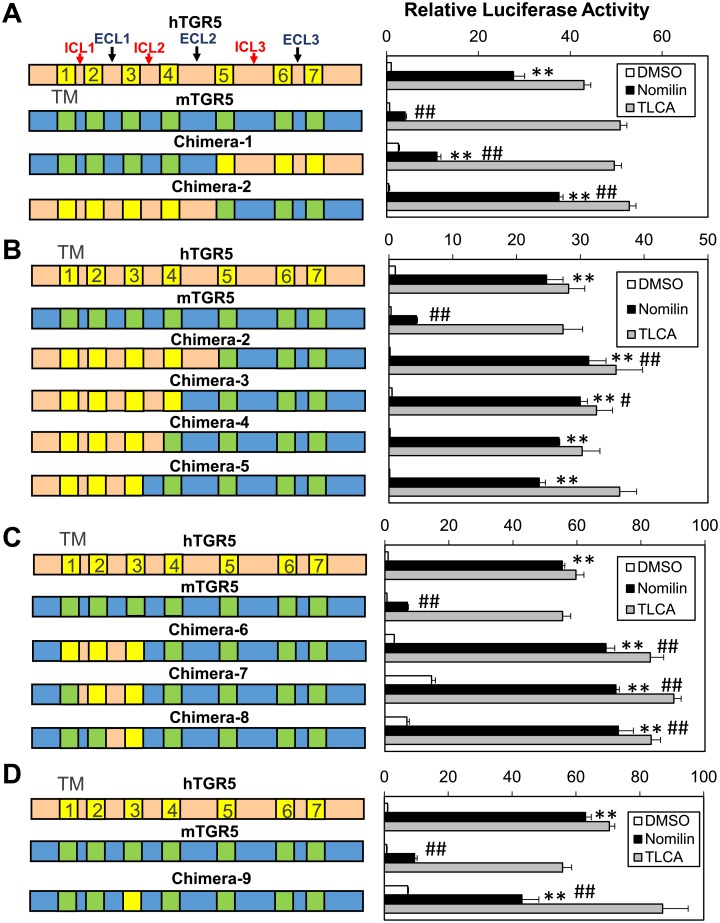
Nomilin and TLCA specificity of chimeric TGR5. (A–D) Left panels show diagrammatic representations of chimeric TGR5. hTGR5 is shown in beige with yellow TM domains, and mTGR5 is shown in blue with green TM domains. Right panels show CRE-Luc response profiles to nomilin and TLCA (100 μM each) for corresponding hTGR5, mTGR5, and chimeric TGR5. Significant differences between the nomilin responses were analyzed using one-way ANOVA (Tukey’s post hoc test); **p < 0.01 for mTGR5-Nomilin; ^#^*p* < 0.05 and ^##^*p* < 0.01 for hTGR5-Nomilin. The values represent the mean ± SD.

To identify candidate amino acid residues that are crucial for nomilin recognition, various human–mouse TGR5 chimeras were developed and analyzed for their responsiveness to TLCA and nomilin. Initially, we constructed two versions of chimeric TGR5: chimera-1 (mTGR5: N-terminal–ECL2; hTGR5: TM5 –C-terminal) and chimera-2 (hTGR5: N-terminal–ECL2; mTGR5: TM5 –C-terminal) (Fig 2A, left panel). A CRE-luciferase reporter assay revealed that chimera-2 exhibits a higher sensitivity to nomilin than does mTGR5 or chimera-1, with an activity comparable to that of hTGR5, suggesting that the N-terminal–ECL2 region in hTGR5 includes crucial sites for nomilin response (Fig 2A). Because the structural and functional hub role of TM3 in class A GPCRs has been confirmed [25], we created three additional TGR5 chimeras with human TM3: chimera-3 (hTGR5: N-terminal–TM4; mTGR5: ECL2 –C-terminal), chimera-4 (hTGR5: N-terminal–ICL2; mTGR5: TM4 –C-terminal), and chimera-5 (hTGR5: N-terminal–TM3; mTGR5: ICL2 –C-terminal) (Fig 2B, left panel). As expected, human-to-mouse replacement of ICL2 –ECL2 exhibited a small effect on the response intensity to nomilin (Fig 2B). Next, CRE-luciferase assay using chimera-6 (mTGR5: N-terminal region, ICL2 –C-terminal; hTGR5: TM1 –TM3), chimera-7 (mTGR5: N-terminal–TM1, ICL2 –C-terminal; hTGR5: ICL1 –TM3), and chimera-8 (mTGR5: N-terminal–TM2, ICL2 –C-terminal; hTGR5: ECL1 –TM3) showed that N-terminal–TM2 of hTGR5 is not necessary for nomilin recognition (Fig 2C). Interestingly, chimera-9 (mTGR5: N-terminal–ECL1, ICL2 –C-terminal; hTGR5: TM3) exhibits a higher response intensity to nomilin than does mTGR5-WT; however, it is lower than that of hTGR5 (Fig 2D). These results suggest that the crucial amino acid residues for nomilin response lie within ECL1 –TM3 in hTGR5.

### Identification of key residues for nomilin response

**Fig 3:**
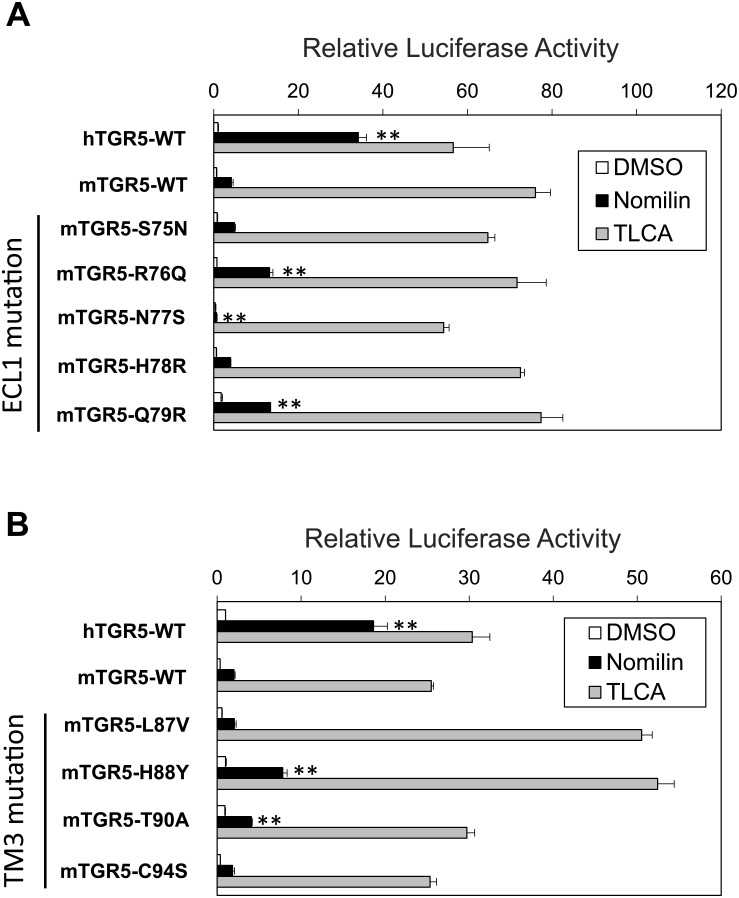
Response profiles of multiple mouse-to-human point mutations in nomilin and TLCA. Transient transfection assays using HEK293 cells with a CRE-luciferase reporter plasmid and expression vector for TGR5 with indicated point mutation of nine unconserved residues in ECL2 (A) and TM3 (B). After transfection for 24 h, the cells were treated with TLCA and nomilin (100 μM each) for another 5 h. Then, luciferase reporter activities were quantified (n = 3). Significant differences between the nomilin responses were analyzed using one-way ANOVA (Tukey’s post hoc test); **p < 0.01 for mTGR5-WT. The values represent the mean ± SD.

Based on the acquired data, nine amino acid residues—N76^ECL1^, Q77^ECL1^, S78^ECL1^, R79^ECL1^, R80^ECL1^, V88^3.28^, Y89^3.29^, A91^3.31^, and S95^3.35^ in hTGR5—were identified as candidate residues for playing a key role in hTGR5–nomilin interaction, which satisfies the following conditions: (1) they are evolutionarily unconserved between hTGR5 and mTGR5, and (2) they exist in ECL1 or TM3. To assess the contribution of these nine residues to nomilin response, nine mouse-to-human one-point mutants were constructed: mTGR5 S75N^ECL1^, R76Q^ECL1^, N77S^ECL1^, H78R^ECL1^, Q79R^ECL1^, L87V^3.28^, H88Y^3.29^, T90A^3.31^, and C94S^3.35^. First, the effects of one-point mutation in ECL1 on nomilin recognition were evaluated using CRE-luciferase reporter assay (Fig 3A). R76Q^ECL1^ and Q79R^ECL1^ showed a significantly higher response to nomilin than that for mTGR5-WT, whereas S75N^ECL1^, N77R^ECL1^, and H78R^ECL1^ showed nearly equal or lower responsiveness to nomilin compared with mTGR5-WT (Fig 3A). Next, we evaluated the effects of one-point mutation in TM3 on nomilin response (Fig 3B). Among these constructs, a CRE-luciferase reporter assay revealed that H88Y^3.29^ and T90A^3.31^ show higher response intensities to nomilin than mTGR5-WT; however, the responsiveness of T90A^3.31^ was weaker than that of H88Y^3.29^ (Fig 3B). L87V^3.28^ and C94S^3.35^ mutants exerted little influence on nomilin response. These results suggest that R76^ECL1^, Q79^ECL1^, and H88^3.29^ in mTGR5 cause low responsiveness to nomilin. Consistent with these findings, human-to-mouse point mutations in these three residues show decreased response intensities to nomilin compared with hTGR5-WT, indicating a crucial role of these three amino acid residues in nomilin recognition by hTGR5-WT (S1 Fig).

### Three amino acid residues make a crucial difference in nomilin response between mTGR5 and hTGR5

**Fig 4:**
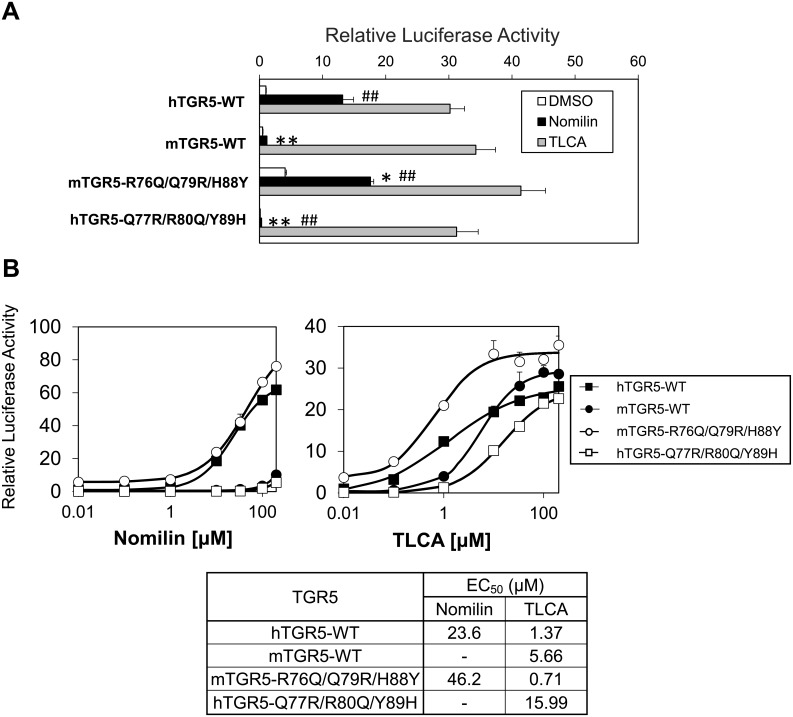
Differences in the affinity between hTGR5 and mTGR5 for nomilin as determined using three amino acid residues. (A) Transient transfection assays using HEK293 cells with a CRE-luciferase reporter plasmid and an expression vector for TGR5 with the indicated mutation of three unconserved residues (n = 3). (B) Each WT and mutant TGR5 was transfected into HEK293 cells together with the CRE-luciferase plasmid, and dose-response curves to nomilin (left panel) and TLCA (right panel) were examined. The lower panel shows the EC_50_ values of each TGR5 for nomilin and TLCA (n = 3). Significant differences between the nomilin responses were analyzed using one-way ANOVA (Tukey’s post hoc test); **p < 0.01 and *p < 0.05 for hTGR5-WT; ^##^*p* < 0.01 and ^#^*p* < 0.05 for mTGR5-WT. The values represent the mean ± SD.

To clarify whether the three amino acid residues account for different nomilin responsiveness between mTGR5 and hTGR5, we constructed mouse-to-human and human-to-mouse triple mutant TGR5 (mTGR5-R76Q^ECL1^/Q79R^ECL1^/H88Y^3.29^ and hTGR5-Q77R^ECL1^/R80Q^ECL1^/Y89H^3.29^). Notably, mTGR5-R76Q^ECL1^/Q79R^ECL1^/H88Y^3.29^ exhibited strong nomilin response equal to that of hTGR5-WT (Fig 4A). By contrast, hTGR5-Q77R^ECL1^/R80Q^ECL1^/Y89H^3.29^ showed a very weak nomilin response identical to that of mTGR5 (Fig 4A). We performed additional luciferase assay experiment with triple alanine-mutated hTGR5 (hTGR5-Q77A^ECL1^/R80A^ECL1^/Y89A^3.29^). As expected, alanine-mutated hTGR5 shows a very weak nomilin response relative to WT-hTGR5 (S2 Fig). On the other hand, alanine-mutated hTGR5 also reduces TLCA responsiveness compared to WT-hTGR5, which is consistent with the previous report showing that hTGR5 Y89A mutant reduces the affinity to bile acids [19].

Next, we calculated half-maximal effective concentration (EC_50_) values from dose-response curves for nomilin and TLCA to measure the affinity of these agonists for human-to mouse and mouse-to human triple mutated TGR5 (Fig 4B). The EC_50_ values of mTGR5-WT and hTGR5-Q77R^ECL1^/R80Q^ECL1^/Y89H^3.29^ mutant for nomilin were undetectable due to the low affinity and solubility of nomilin at higher concentration, whereas mTGR5-R76Q^ECL1^/Q79R^ECL1^/H88Y^3.29^ showed EC_50_ values similar to those of hTGR5-WT (mTGR5-R76Q^ECL1^/Q79R^ECL1^/H88Y^3.29^, 46.2 μM; hTGR5-WT, 23.6 μM) (Fig 4B). The EC_50_ values of TLCA were also altered due to the triple mutations; however, these changes were not as evident as those for nomilin (hTGR5-WT, 1.37 μM; mTGR5-WT, 5.66 μM; mTGR5-R76Q^ECL1^/Q79R^ECL1^/H88Y^3.29^, 0.71 μM; hTGR5-Q77R^ECL1^/R80Q^ECL1^/Y89H^3.29^, 15.99 μM) (Fig 4B). Taken together, we deduced that Q77^ECL1^, R80^ECL1^, and Y89^3.29^ are key amino acid residues that determine the high affinity of nomilin to hTGR5.

**Fig 5:**
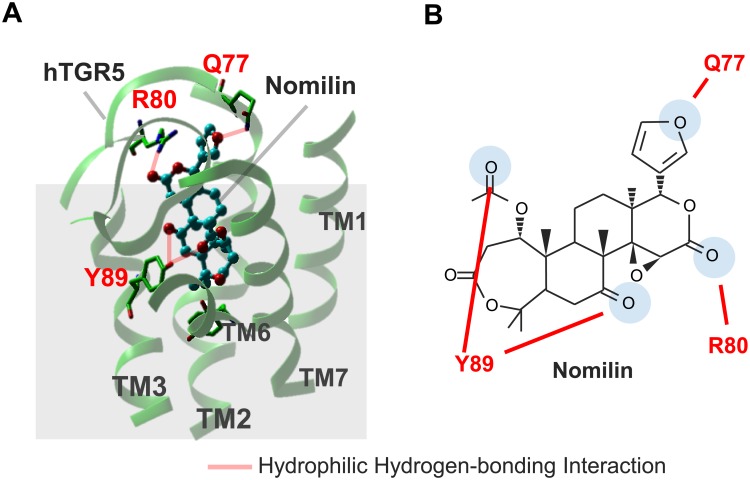
Binding modes and docking interactions of nomilin with hTGR5. (A and B) Hydrophilic hydrogen-bonding interaction between nomilin and three amino acid residues of hTGR5 are shown with red dashes.

On the basis of these findings, we constructed an hTGR5–nomilin binding model in silico. Because the structural model of nomilin-bound hTGR5 should be constructed from its activated form, we selected metarhodopsin II [21], one of the few GPCRs whose active form structures were solved, as a structural template (Fig 5A). The crystal structure of the transmembrane regions of metarhodopsin structure bound to C-terminal peptide of transducin, a G protein shows a good agreement with an activated structure of the adrenergic receptor which binds G protein at the C-terminus peptide region. Thus, the present study used the structure of metarhodopsin II for the construction of the complex models of TGR5 bound to the limonoid-related derivatives. Within two binding modes examined for the complex model, the mode which showed better agreement with the mutational data was selected as an appropriate binding mode. As shown in Fig 5A and 5B, nomilin is estimated to play a role in four hydrophilic hydrogen-bonding interactions with Q77^ECL1^, R80^ECL1^, and Y89^3.29^ (shown in red-dashed line), resulting in the activation of hTGR5. The binding of nomilin in this study showed that it binds in the mode vertical to the membrane. This mode is different from that in the complexes reported by Macchiarulo et al in which rhodopsin, an inactive GPCR, was used as the template for the TGR5 receptor [19]. This may reflect the difference of their structures at A-ring and furan portions. The limonoid derivatives have bulkier moieties at both edges of the molecules than those of bile acid derivatives.

### Obacunone exhibits different agonist activity between hTGR5 and mTGR5 in a manner similar to nomilin

**Fig 6:**
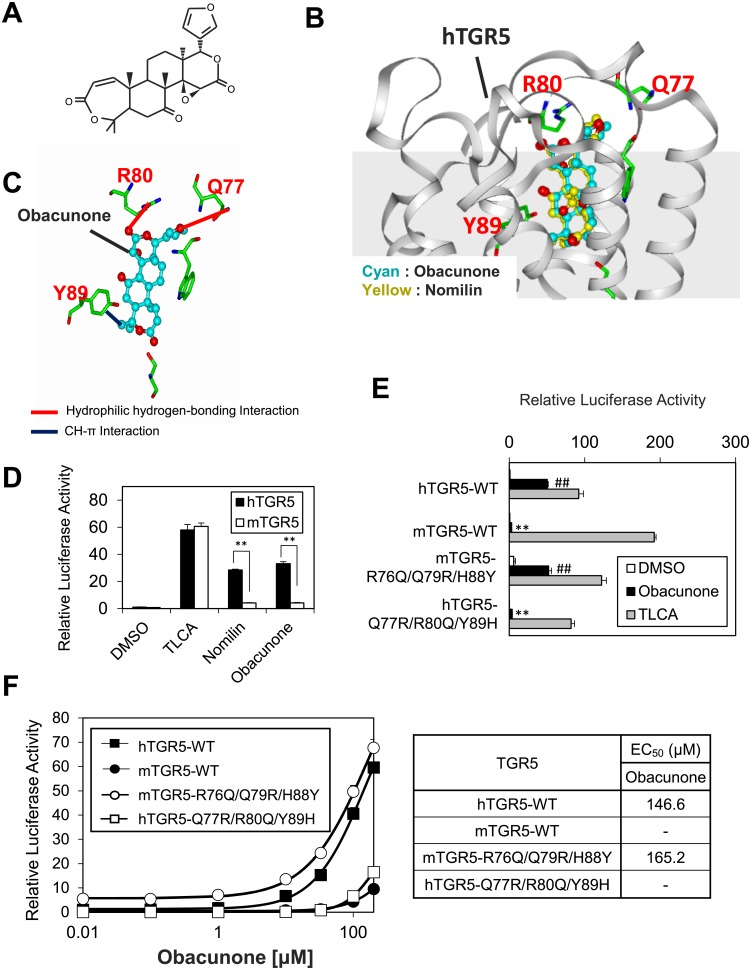
Obacunone activates hTGR5 in a binding pattern analogous to nomilin. (A) Structural representation of obacunone. (B) Comparison between the binding pattern of hTGR5 and those of obacunone (cyan) and nomilin (yellow). (C) Hydrophilic hydrogen-bonding interaction and CH-π interaction occurring between obacunone and three amino acid residues of hTGR5 are shown in red- and blue-dashed lines, respectively. (D) HEK293 cells were transfected with the CRE-driven luciferase reporter plasmid and the hTGR5 or mTGR5 expression plasmid. After transfection for 24 h, the cells were treated with TLCA, nomilin, and obacunone (100 μM each) for another 5 h. Then, the luciferase reporter activity was quantified. Significant differences were analyzed using one-way ANOVA (Tukey’s post hoc test); **p < 0.01. (E) Response profiles to DMSO, obacunone, and TLCA indicated for TGR5. Significant differences between the obacunone responses were analyzed using one-way ANOVA (Tukey’s post hoc test); **p < 0.01 for hTGR5-WT; ^##^*p* < 0.01 for mTGR5-WT. (F) Each WT and mutant TGR5 was transfected into HEK293 cells together with CRE-luciferase plasmid, and the dose-response curves to obacunone were examined. The right panel shows the EC_50_ of each TGR5 for the obacunone. The values represent the mean ± SD.

Although we previously identified obacunone, a nomilin derivative, as a TGR5 agonist, the binding mode of obacunone–TGR5 has not been clarified [16, 26]. Because obacunone is structurally related to nomilin, sharing the three oxygen atoms that form hydrophilic hydrogen-bonding interactions with Q77^ECL1^, R80^ECL1^, and Y89^3.29^ in hTGR5 (Figs 5A, 5B and 6A), we examined whether obacunone and hTGR5 form a binding mode similar to that of nomilin and hTGR5. A docking simulation showed that obacunone appears to bind to the same position of hTGR5 as nomilin (Fig 6B), forming two hydrophilic hydrogen-bonding interactions and one CH-π interaction with Q77^ECL1^, R80^ECL1^, and Y89^3.29^ (Fig 6C). If this binding model is correct, obacunone is expected to exhibit different agonist activity between hTGR5 and mTGR5. Indeed, obacunone exhibits stronger agonist activity for hTGR5 than it does for mTGR5, and this response pattern is quite similar to nomilin (Fig 6D). To confirm the role of Q77^ECL1^, R80^ECL1^, and Y89^3.29^ in obacunone–hTGR5 interaction, we examined the obacunone responsiveness of hTGR5-WT, mTGR5-WT, mTGR5-R76Q^ECL1^/Q79R^ECL1^/H88Y^3.29^, and hTGR5-Q77R^ECL1^/R80Q^ECL1^/Y89H^3.29^. As expected from the obacunone–hTGR5 binding model, hTGR5-WT and mTGR5-R76Q^ECL1^/Q79R^ECL1^/H88Y^3.29^ showed equally intense obacunone responses in contrast with that of mTGR5-WT and hTGR5- Q77R^ECL1^/R80Q^ECL1^/Y89H^3.29^, which showed a very weak obacunone response (Fig 6E). Next, EC_50_ values were calculated from dose-response curves for obacunone (Fig 6F). As in the case of nomilin, the EC_50_ values of mTGR5-WT and hTGR5-Q77R^ECL1^/R80Q^ECL1^/Y89H^3.29^ mutant for obacunone were undetectable, and hTGR5-WT and mTGR5- R76Q^ECL1^/Q79R^ECL1^/H88Y^3.29^ showed similar EC_50_ values (146.6 μM and 165.2 μM, respectively) (Fig 6F). These results suggest that both obacunone and nomilin form a similar binding mode with hTGR5 and that Q77^ECL1^, R80^ECL1^, and Y89^3.29^ are essential for these interactions.

Another docking simulation predicted that limonin could not activate hTGR5 in contrast to the TGR5–nomilin/obacunone interaction because the 6-membered ring lactone and furan ring in this molecule form steric repulsion with W75^ECL1^ and S78^ECL1^ (S3A Fig). Indeed, limonin exhibited very weak hTGR5 agonist activity [16] (S3B Fig).

## Discussion

Because GPCRs comprise the largest class of human membrane proteins, which mediate many physiological processes, they are recognized as major drug targets [27]. Recently, *in silico* methods such as pharmacophore modelling and molecular dynamics simulation have rapidly advanced, enabling fast and easy screening for identifying new GPCR agonists and antagonists. Indeed, some TGR5 agonists were identified using pharmacophore modeling [28, 29]. However, structural information on TGR5–ligand interaction is insufficient for highly accurate *in silico* screening, causing low predictive power. Therefore, we aimed to clarify molecular details playing a role in the interaction between TGR5 and nomilin, which is a naturally occurring TGR5 agonist.

Notably, a CRE-luciferase reporter assay using hTGR5 and mTGR5 clearly showed that hTGR5 shows a higher response to nomilin than does mTGR5 (Fig 1B). On the basis of this finding, we generated several types of expression plasmids for mouse–human chimeric TGR5 and revealed the important region for hTGR5–nomilin interaction (Fig 2A–2D). In addition, using mouse-to-human point-mutated TGR5, three amino acid residues (Q77^ECL1^, R80^ECL1^, and Y89^3.29^ in hTGR5) were identified as key residues for nomilin response (Fig 3A and 3B). In general, class A GPCRs, including TGR5, show a consensus ligand-binding pocket, and TM3 is the hub for this structure [25]. This supports our finding that hTGR5 Y89^3.29^ is one of the critical residues involved in the hTGR5–nomilin interaction. Another report showed that positively charged residues in ECL1 could play an important role in agonist binding, which is consistent with the fact that hTGR5 R80^ECL1^ contributes to nomilin binding [30]. Furthermore, hTGR5-WT and mTGR5-R76Q^ECL1^/Q79R^ECL1^/H88Y^3.29^ showed EC_50_ values similar to those of nomilin, whereas those of mTGR5-WT and hTGR5-Q77R^ECL1^/R80Q^ECL1^/Y89H^3.29^ were lower (Fig 4B). Changes in the EC_50_ values of TLCA due to these triple mutations were relatively lower than those in the EC_50_ values of nomilin. These results confirmed that Q77^ECL1^, R80^ECL1^, and Y89^3.29^ are crucial for the hTGR5–nomilin interaction and that this binding pattern is different from that for TLCA (Fig 4B).

Based on these results, the hTGR5–nomilin docking model was constructed (Fig 5A). Because nomilin is a TGR5 agonist, we selected metarhodopsin II, an active form of rhodopsin, as a template [21, 31]. The docking simulation showed that hTGR5 Q77^ECL1^, R80^ECL1^, and Y89^3.29^ form four hydrophilic hydrogen-bonding interactions with four oxygen atoms in nomilin (Fig 5B). Obacunone, a limonoid sharing three oxygen atoms with nomilin, which leads to hydrophilic hydrogen-bonding interactions with Q77^ECL1^, R80^ECL1^, and Y89^3.29^ (Fig 6A), also exhibited higher agonist activity for hTGR5 than for mTGR5 (Fig 6D). In addition, our triple mutation study showed that Q77^ECL1^, R80^ECL1^, and Y89^3.29^ were crucial for hTGR5–obacunone interaction, akin to nomilin (Fig 6E and 6F). The docking simulation also indicated that limonin could not activate hTGR5 in a manner identical to nomilin or obacunone due to the steric repulsion caused by a 6-membered ring lactone and furan ring (S3A Fig). These results support the relevance of hTGR5–nomilin binding modes as determined using *in silico* docking simulation. A previous report showed that TLCA forms a salt bridge and two hydrogen bonds with R79^EL1^, E169^5.44^, and Y240^6.51^, suggesting that TLCA bind to the wider space in the hTGR5 ligand binding pocket than nomilin [20]. Higher efficacies of TLCA relative to nomilin might be partially explained by the difference of these binding positions.

In conclusion, we identified the key amino acid residues and molecular features of the hTGR5–nomilin interaction. The binding mode of hTGR5–nomilin is vastly different from that of other TGR5 agonists, such as TLCA and INT-777 [19, 20], suggesting that TGR5 forms various binding patterns depending on the agonist type. Our study promotes a better understanding of the structure of TGR5, and it may help in the future development and screening of new TGR5 agonists.

## Supporting information

S1 Fig The response profiles of the multiple human-to-mouse point mutations to nomilin and TLCA. Transient transfection assays using HEK293 cells with a CRE-luciferase reporter plasmid and an expression vector for TGR5 with indicated point mutation. Twenty-four hours after transfection, cells were treated with TLCA or nomilin (100 μM each) for another 5 h. Then, luciferase reporter activity was quantified (hTGR5/DMSO was set at 1) (n = 3). Significant differences between the nomilin responses were analyzed using one-way ANOVA (Tukey’s post hoc test) (*p < 0.05; **p < 0.01 for hTGR5). The values represent the mean ± SD.
(PDF)

S2 Fig Response profile of triple alanine mutations in nomilin and TLCA. Transient transfection assays using HEK293 cells with a CRE-luciferase reporter plasmid and an expression vector for triple alanine mutated TGR5. Twenty-four hours after transfection, cells were treated with TLCA or nomilin (100 μM each) for another 5 h. Then, luciferase reporter activity was quantified (hTGR5/DMSO was set at 1) (n = 3). Significant differences in nomilin treated group were analyzed using one-way ANOVA (Tukey’s post hoc test) (*p < 0.05; **p < 0.01 for hTGR5). The values represent the mean ± SD.
(PDF)

S3 Fig Binding modes and activation potency of limonin for hTGR5. (A) Comparison of binding pattern of hTGR5 and limonin (cyan). Steric repulsion formed between limonin and hTGR5 is indicated by red double-headed arrows. (B) HEK293 cells were transfected with the CRE-driven luciferase reporter plasmid and the hTGR5 expression plasmid. After transfection for 24 h, the cells were treated with TLCA (positive control) and limonin (100μM each) for another 5 h. Then, luciferase reporter activity was quantified (hTGR5 (-)/DMSO was set at 1) (n = 3). Significant differences were analyzed using one-way ANOVA (Tukey’s post hoc test); **p < 0.01. The values represent the mean ± SD.
(PDF)

S1 File Construction of chimera and mutated TGR5. (PDF)
