# Supplementary figures and images for: Identification of key amino acid residues in the hTGR5–nomilin interaction and construction of its binding model
Source: PLoS One. 2017 Jun 8;12(6):e0179226. doi: 10.1371/journal.pone.0179226 (PMC5464637; doi:10.1371/journal.pone.0179226)

## Relative Luciferase Activity

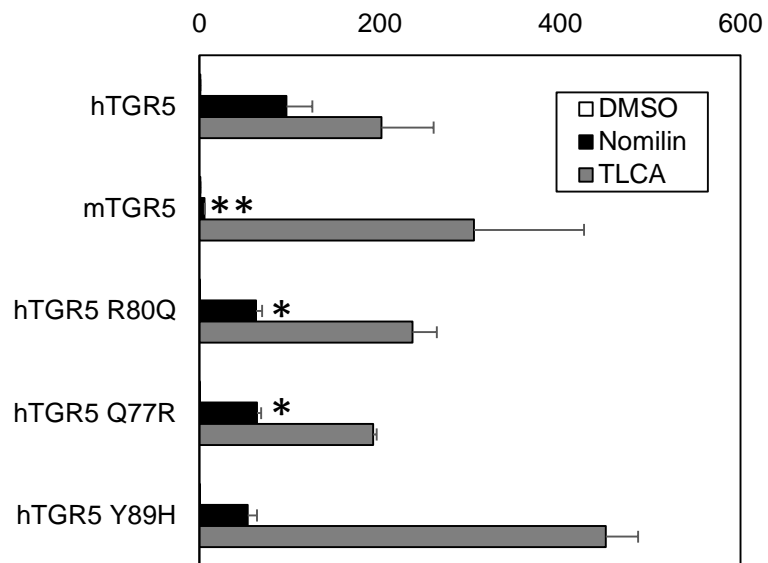

Supplement: S1 Fig — Transient transfection assays using HEK293 cells with a CRE-luciferase reporter plasmid and an expression vector for TGR5 with indicated point mutation. Twenty-four hours after transfection, cells were treated with TLCA or nomilin (100 μM each) for another 5 h. Then, luciferase reporter activity was quantified (hTGR5/DMSO was set at 1) (n = 3). Significant differences between the nomilin responses were analyzed using one-way ANOVA (Tukey’s post hoc test) (*p < 0.05; **p < 0.01 for hTGR5). The values represent the mean ± SD. (PDF) [file pone.0179226.s001.pdf]

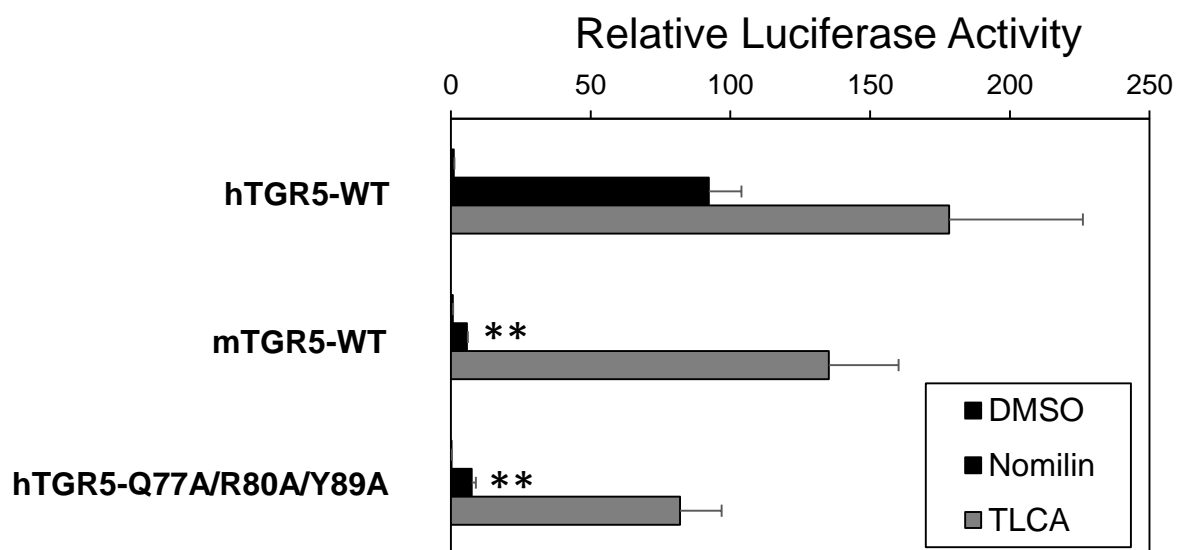

Supplement: S2 Fig — Transient transfection assays using HEK293 cells with a CRE-luciferase reporter plasmid and an expression vector for triple alanine mutated TGR5. Twenty-four hours after transfection, cells were treated with TLCA or nomilin (100 μM each) for another 5 h. Then, luciferase reporter activity was quantified (hTGR5/DMSO was set at 1) (n = 3). Significant differences in nomilin treated group were analyzed using one-way ANOVA (Tukey’s post hoc test) (*p < 0.05; **p < 0.01 for hTGR5). The values represent the mean ± SD. (PDF) [file pone.0179226.s002.pdf]

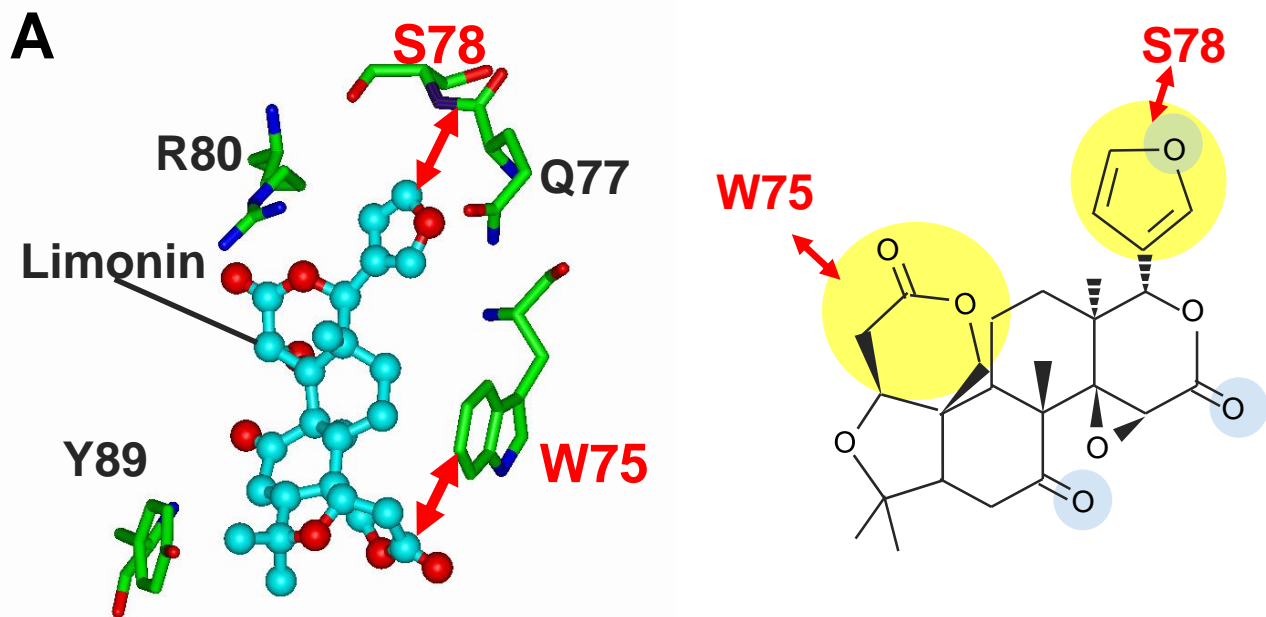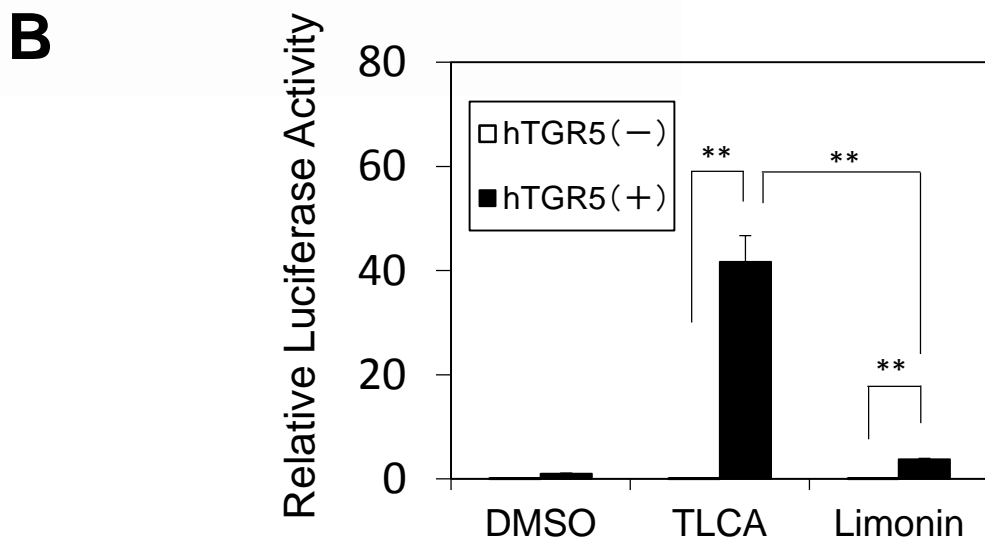

Supplement: S3 Fig — (A) Comparison of binding pattern of hTGR5 and limonin (cyan). Steric repulsion formed between limonin and hTGR5 is indicated by red double-headed arrows. (B) HEK293 cells were transfected with the CRE-driven luciferase reporter plasmid and the hTGR5 expression plasmid. After transfection for 24 h, the cells were treated with TLCA (positive control) and limonin (100μM each) for another 5 h. Then, luciferase reporter activity was quantified (hTGR5 (-)/DMSO was set at 1) (n = 3). Significant differences were analyzed using one-way ANOVA (Tukey’s post hoc test); **p < 0.01. The values represent the mean ± SD. (PDF) [file pone.0179226.s003.pdf]
