# Supplementary material for: Identification of key amino acid residues in the hTGR5–nomilin interaction and construction of its binding model
Source: PLoS One. 2017 Jun 8;12(6):e0179226. doi: 10.1371/journal.pone.0179226 (PMC5464637; doi:10.1371/journal.pone.0179226)
Supplement: S1 File — (PDF) [file pone.0179226.s004.pdf]

Table A. Construction of Chimera-1 and-2.

chimera-1	mTGR5(3-168)	F	HindIII	5’-ATAT AAGCTT ACA CCC AAC AGC ACT GAG CTG-3’
		R	XbaI	5’-ATAT TCTAGA TAGAGGTAGGGGGCT-3’
	hTGR5(167-330)	F	XbaI	5’-ATAT TCTAGA AGTCTATGGGCTCCT-3’
		R	BamHI	5’-ATAT GGATCC TTA GTT CAA GTC CAG GTC GAC ACT GC-3’

chimera-2	hTGR5(2-169)	F	HindIII	5’-ATAT AAGCTT ACG CCC AAC AGC ACT GGC GAG-3’
		R	XbaI	5’-ATAT TCTAGA TAGAGGTAGGGGGCT-3’
	mTGR5(166-329)	F	XbaI	5’-ATAT TCTAGA AGTTTATGGCCTCCT-3’
		R	BamHI	5’-ATAT GGATCC CTA ATT CAA GTC CAG GTC AAT GCT G-3’

Template: WT-hTGR5, WT-mTGR5

Table B. Construction of Chimera-3 to -5

Forward primer (h) : 5’-ATAT AAGCTT ACGCCCAACAGCACTGGCGAG -3’
Reverse primer (m) : 5’-ATAT GGATCC CTAATTCAAGTCCAGGTCAATGCTG -3’

Chimera-3	Forward	5’-GCCCGCTCTGGGCTGGAAC-3’
	Reverse	5’-GGTTCCAGCCCAGAGCGG-3’

Chimera-4	Forward	5’-GCCCCCTGGGAGTGTGCG-3’
	Reverse	5’-GCCGCACACTCCCAGGGG-3’

Chimera-5	Forward	5’-CTTGCTGGTGCATGGGGAAC-3’
	Reverse	5’-CGTTCCCCATGCACCAGCAAG-3’

Template: WT-hTGR5, WT-mTGR5

Table C. Construction of Chimera-6

Chimera-6	Forward	5'-ATATAAGCTTACACCCAACAGCACTGAGCTGTCGG CCATTCCCATGGGGGTTCTGGGGCTCTCCCTG-3'
	Reverse (m)	5'-ATATGGATCCCTAATTCAAGTCCAGGTCAATGCTG-3'

Template: Chimera-5

Table D. Construction of Chimera-7 to -9.

Forward primer (m) : 5'-ATATAAGCTTACACCCAACAGCACTGAGCTG -3'
Reverse primer (m) : 5'-ATATGGATCCCTAATTCAAGTCCAGGTCAATGCTG -3'

Chimera-7	Forward	5'-GCCCTGGACCGCCGCCTG -3'
	Reverse	5'-CAGGCGGCGGTCCAGGGC-3'
Chimera-8	Forward	5'-CATGCTGCCTGGGCTGTGG -3'
	Reverse	5'-CCACAGCCCAGGCAGCATG-3'
Chimera-9	Forward	5'-GGGCTACTGGTCCTGCCTCC-3'
	Reverse	5'-GGAGGCAGGACCAGTAGCCC-3'

Template: WT-mTGR5, Chimera-5

Table E. Primer set for mouse-to-human point mutated TGR5

HindⅢ	F	5’-ATAT AAGCTT ACA CCC AAC AGC ACT GAG CTG-3’
BamHI	R	5’-ATAT GGATCC CTA ATT CAA GTC CAG GTC AAT GCT G-3’

S75N	Forward	5’-GGGCTATGGAACCGGAACCATC-3’
	Reverse	5’-GATGGTTCCGGTTCCATAGCCC-3’
R76Q	Forward	5’-GCTATGGAGCCAGAACCATCAGG-3’
	Reverse	5’-CCTGATGGTTCTGGCTCCATAGC-3’
N77S	Forward	5’-CTATGGAGCCGGAGTCATCAGGGC-3’
	Reverse	5’-GCCCTGATGACTCCGGCTCCATAG-3’
H78R	Forward	5’-GAGCCGGAACCGCCAGGGCTACTG-3’
	Reverse	5’-CAGTAGCCCTGGCGGTTCCGGCTC-3’
Q79R	Forward	5’-CGGAACCATCGGGGCTACTGG-3’
	Reverse	5’-CCAGTAGCCCCGATGGTTCCG-3’
L87V	Forward	5’-GGTCCTGCCTCCTTGTCCACTTGACCCCCAACTTTTG-3’
	Reverse	5’-CAAAAGTTGGGGGTCAAGTGGACAAGGAGGCAGGACC-3’
H88Y	Forward	5’-GGTCCTGCCTCCTTCTCTACTTGACCCCCAACTTTTG-3’
	Reverse	5’-CAAAAGTTGGGGGTCAAGTAGAGAAGGAGGCAGGACC-3’
T90A	Forward	5’-CTCCACTTGGCTCCCAACTTTTG-3’
	Reverse	5’-CAAAAGTTGGGAGCCAAGTGGAG-3’
C94S	Forward	5’-CCCAACTTTTCCTTCCTTTCCCTG-3’
	Reverse	5’-CAGGGAAAGGAAGGAAAAGTTGGG-3’

Table F. Primer set for human-to-mouse point mutated TGR5

HindⅢ	F	5’-ATAT AAGCTT ACG CCC AAC AGC ACT GGC GAG-3’
BamHI	R	5’-ATAT GGATCC TTA GTT CAA GTC CAG GTC GAC ACT GC-3’

Y89H	Forward	5’-CCTGCCTCCTCGTCCACTTGGCTCCCAACTTC-3’
	Reverse	5’-GAAGTTGGGAGCCAAGTGGACGAGGAGGCAGG-3’
Q77R	Forward	5’-CTGTGGAACCGGAGTCGCCG-3’
	Reverse	5’-CGGCGACTCCGGTTCCACAG-3’
R80Q	Forward	5’-CAGAGTCGCCAGGGTTACTGG-3’
	Reverse	5’-CCAGTAACCCTGGCGACTCTG-3’
